# Can you catch Ebola from a stork bite? Inductive reasoning influences generalization of perceived zoonosis risk

**DOI:** 10.1371/journal.pone.0186969

**Published:** 2017-11-08

**Authors:** Tyler Davis, Micah B. Goldwater, Molly E. Ireland, Nicholas Gaylord, Jason Van Allen

**Affiliations:** 1 Department of Psychological Sciences, Texas Tech University, Lubbock, Texas, United States of America; 2 School of Psychology, University of Sydney, Sydney, New South Wales, Australia; 3 Independent Research Consultant, San Francisco, California, United States of America; University of Washington, UNITED STATES

## Abstract

Emerging zoonoses are a prominent global health threat. Human beliefs are central to drivers of emerging zoonoses, yet little is known about how people make inferences about risk in such scenarios. We present an inductive account of zoonosis risk perception, suggesting that beliefs about the range of animals able to transmit diseases to each other influence how people generalize risks to other animals and health behaviors. Consistent with our account, in Study 1, we find that participants who endorse higher likelihoods of cross-species disease transmission have stronger intentions to report animal bites. In Study 2, using real-world descriptions of Ebola virus from the WHO and CDC, we find that communications conveying a broader range of animals as susceptible to the virus increase intentions to report animal bites and decrease perceived safety of wild game meat. These results suggest that inductive reasoning principles may be harnessed to modulate zoonosis risk perception and combat emerging infectious diseases.

## Introduction

Emerging infectious diseases are major economic and public health concerns. A majority of such diseases are of zoonotic origin [1, 2]. Drivers of emerging zoonoses include consumption of wild game meat, livestock grazing practices, and adverse contact with animals through bites and handling of carcasses [3, 4]. For all of these drivers, human-animal interaction plays a critical role in disease emergence, and thus investigating the factors that influence human risk perception is critical to prevention of future pandemics. However, the factors that determine how people reason about zoonosis risks and generalize such knowledge to novel scenarios remain under studied [5].

Research on zoonosis risk perception has uncovered a number of person-level factors and beliefs that influence people’s perceptions of zoonosis risk and their intentions to take preventative measures. Like other areas of risk perception in public health [6], people’s perception of zoonotic risks and their efforts to take preventative behaviors are influenced by their perceptions of personal vulnerability, disease severity, self-efficacy with respect to taking appropriate preventative measures, and perceived response efficacy of the behavior [7, 8]. Other work has focused more exclusively on demographic variables and beliefs associated with specific diseases, disease reservoirs, and risky behaviors in at risk populations (for review and commentary, see [9]) without connecting these into a broader theoretical or psychological framework [7]. For example, a recent study of risk perception in Ghana found that people are aware of bats as reservoirs of Ebola and other zoonoses, but are less interested in factors that may mitigate their risks such as post-exposure prophylaxis, washing fruits, or controlling the bat population [10]. Other recent work has found age, gender, and urbanization to influence perceptions of bat risk and intentions to eat bats [11].

The focus of most zoonosis risk perception research on person-level variables and knowledge or beliefs about specific diseases raises the question of how people generalize knowledge about diseases to novel scenarios. Indeed, when dealing with diseases that are novel, or new to a particular region or group, inferences that people make about possible reservoirs, protective behaviors, and personal risk may largely be governed by their knowledge of completely different diseases. Likewise, knowledge about particular reservoirs or safety practices often needs to be generalized to novel animals because health communications rarely enumerate the full range of susceptible animals or drivers. Thus, in order to effectively manage zoonosis risk, individuals often need to generalize their knowledge to make inferences about how their previous experiences and beliefs apply in unknown situations.

Although not a focus of zoonosis risk perception research, how people generalize information to novel scenarios is a main area of inductive reasoning research in cognitive psychology. Inductive reasoning is the practice of generalizing beliefs or knowledge from previous experiences and known examples to novel situations. For example, in terms of zoonosis risk perception, people may reason inductively from their past experience or knowledge that because dogs can catch rabies and transmit it to humans, cats and other mammals may be susceptible to rabies as well. Likewise, in terms of perceptions of Ebola risk, people who know that bats are reservoirs for Ebola may wonder about the risks associated with other small mammals (e.g., rodents) or even animals that share a superficial similarity (e.g., having wings), such as storks. A hallmark of inductive reasoning research is that knowledge about one species of animal, for example, can influence beliefs about very different animals [12–14].

Despite there being little research on generalization in the zoonosis risk perception literature, there has been some evidence to suggest that people’s beliefs about one type of animal can influence their beliefs about different species. For example, one recent study found that survey respondents were more likely to report dog bites if they knew that bats could transmit rabies to humans [15]. These results are surprising from the perspective of current zoonosis risk perception frameworks—people’s inferences about the risks posed by one species (dogs) appear to be influenced by their knowledge of a completely different species (bats). However, the idea that people may generalize knowledge about bats to other mammals is anticipated by cognitive research on inductive reasoning.

Bingham et al.’s [15] finding that knowledge about one animal (bats) can affect beliefs about risks associated with other animals (dogs) may be partly accounted for by two cognitive principles from research on how people make inductive inferences: *premise number* and *premise diversity* [13, 14]. According to the premise number principle, people are more confident in inferences that apply to a large number of category members [16, 17]. For example, participants told that lions *and* giraffes use norepinephrine as a neurotransmitter will tend to judge rabbits as more likely to use norepinephrine than those told that only lions *or* giraffes do so. In terms of zoonosis risk, knowing that multiple mammals can transmit a disease to humans may increase the perceived likelihood of humans contracting that disease from another animal’s bite. According to the premise diversity principle, people find inferences sound to the extent that they are known to hold for a wider range of category members [18, 19]. For example, participants told that *lions and giraffes* use norepinephrine as a neurotransmitter will tend to judge a generalization to rabbits as stronger than participants told that *lions and tigers* use norepinephrine as a neurotransmitter. In terms of zoonosis risk, knowing that both dogs and bats can transmit rabies may increase perceptions of human risk because they are often viewed as very different members of the mammal category.

Although premise number and diversity are plausibly related to the previous observations surrounding bite reporting intentions, research on inductive reasoning has not been extended to work on risk perception in health or real-world decision making about health behaviors. Indeed, basic research on induction often focuses on the underlying cognitive mechanisms by abstracting beyond applications in any particular domain. Moreover, people’s risk perceptions and judgments of infectious disease contagion have been studied through a psychological lens [20–24], but, like a majority of zoonosis perception research, these studies have focused on person-level knowledge, affective reactions, and cultural influences on transmission beliefs rather than fine-grained inductive principles related to generalization of risk and contagion beliefs.

In the present work, we test two specific hypotheses from our theory that inductive reasoning principles influence zoonosis risk perception. First, consistent with the aforementioned rabies study, individual differences in perceived human risk from animal contact should be associated with individual differences in beliefs about interspecies disease transmission. Second, communications depicting disease transmission to humans from a wider range of species should increase perceptions of risks posed by other animals and the perceived human risk of contact.

## Study 1

The goal of Study 1 was to examine whether intentions to report animal bites are associated with beliefs about interspecies disease transmission. Specifically, based on the premise number principle, we hypothesized that individuals who endorse stronger likelihoods of disease transmission between a number of different animal species would be more likely to generalize this risk to humans and thus perceive greater risks from animal bites. To test this hypothesis, we conducted a survey measuring intentions to report bites from a number of common mammals and birds along with judgments of interspecies disease transmission likelihood for a fictitious novel disease.

### Methods

**Fig 1:**
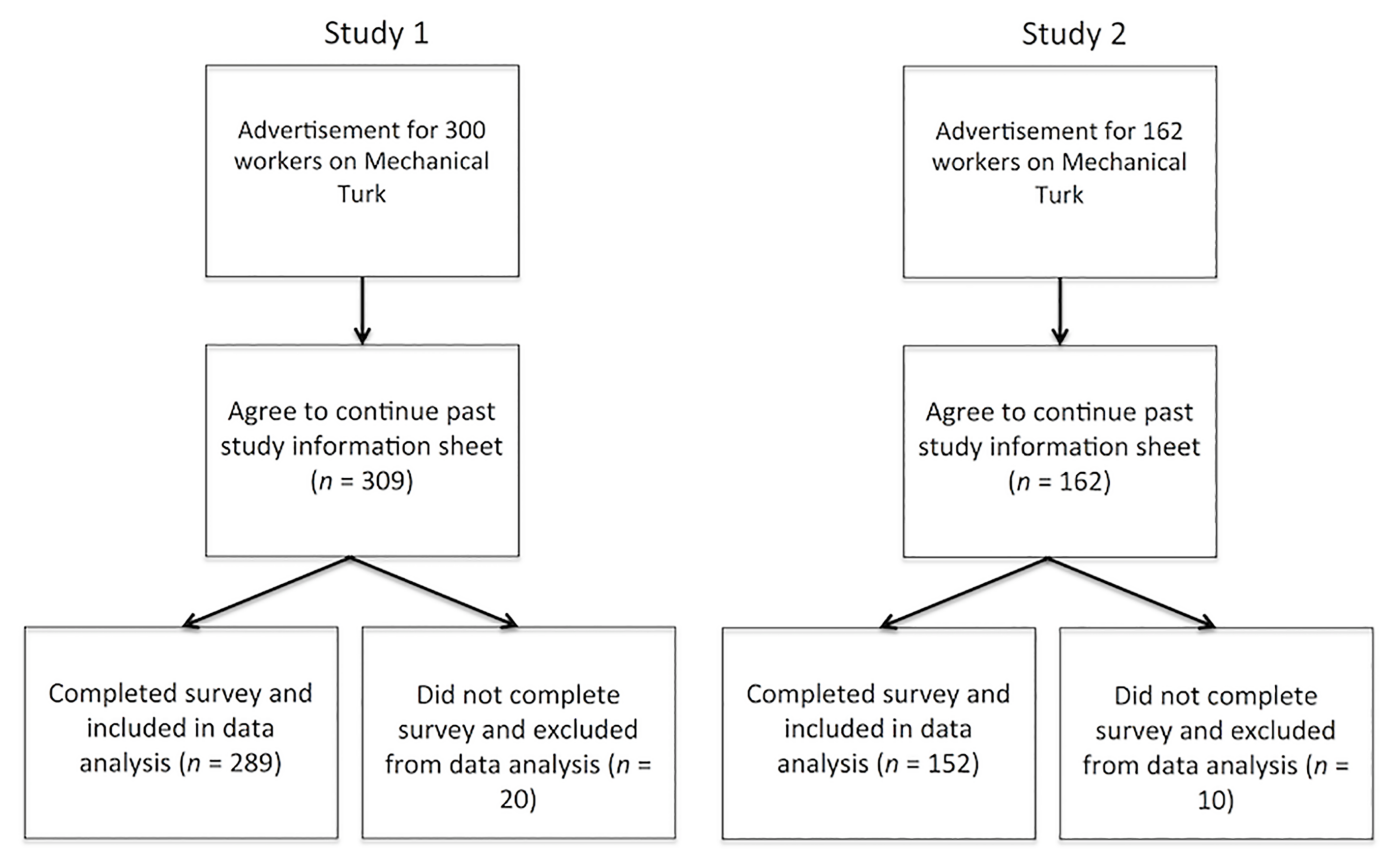
Recruitment diagram. Excluded participants are those who exited the survey window prior to its completion.

Participants were 289 adults recruited from Amazon’s Mechanical Turk between May 18, 2015 and May 26, 2015 to participate in a cross-sectional study on inductive reasoning (See Table 1 for demographics and Fig 1 for recruitment diagram). Mechanical Turk is an online crowd-sourcing platform that has become popular as a recruitment tool for social and cognitive psychology studies [24, 25] and clinical research [26]. The online survey was available to Mechanical Turk workers in the following countries where English is the primary language: USA, Australia, Canada, Great Britain, Ireland, New Zealand, and the Bahamas. Participants self-selected based on an advertisement offering $2 compensation for completing a survey entitled “Texas Tech Animal Categories 1.” Eligibility criteria listed in the advertisement included being at least 18 years of age, being able to write and speak English fluently, and not having completed the study previously. The survey was described as investigating how people reason about categories, such as animals, objects, activities, scenes, or foods. The advertisement had a link that redirected interested participants to the survey hosted on Qualtrics. The first page of the survey contained an information sheet with additional details about the study, potential risks, confidentiality, and contact information for the PI. Participants decided whether or not to participate by reading the information sheet and proceeding with the study. Agreement to participate was recorded based on whether the participant proceeded beyond the information sheet. Written consent was not required due to exempt classification and the low risk and anonymous online nature of the survey data. The consent process and study protocols were approved by the Institutional Review Board of Texas Tech University. Participants were excluded from all analyses if they closed the survey prior to its end. All survey materials, analyses, and data are available at http://osf.io/4r79f

**Table 1:** Demographic characteristics in Studies 1 and 2.

	Study 1 *N* (%)	Study 2 *N* (%)
*Respondents*^a^		
Complete	289 (94.5)	152 (93.8)
Incomplete	20 (6.5)	10 (6.2)
*Age*		
Range	19–75	19–68
Median	31	32
Mean (*SD*)	33.5 (10.2)	34.1 (9.5)
*Sex*		
Male	159 (55.0)	75 (49.3)
Female	130 (45.0)	76 (50.0)
Prefer not to say	0 (0)	1 (< .1)
*Sexual Orientation*		
Straight or heterosexual	260 (90.0)	137 (90.1)
Gay or homosexual	7 (2.4)	4 (2.6)
Bisexual	15 (5.2)	8 (5.3)
Other	3 (1.0)	1 (< .1)
Prefer not to say	5 (1.4)	2 (1.3)
*Political Orientation*		
Very liberal	61 (21.1)	22 (14.5)
Somewhat liberal	101 (34.9)	59 (38.8)
Neither	71 (24.6)	29 (19.1)
Somewhat conservative	45 (15.6)	32 (21.1)
Very conservative	11 (3.8)	10 (6.6)
*Ethnicity*		
Asian	7 (5.9)	15 (9.9)
Black	11 (3.8)	9 (5.9)
Hispanic	20 (6.9)	2 (1.3)
Native American or Alaskan Native	3 (1.0)	1 (< .1)
White	233 (80.6)	121(79.6)
Other	4 (1.4)	3 (2.0)
Prefer not to say	0 (0)	1 (< .1)
*Education*		
Some middle	0 (0)	0 (0)
Some high-school	19 (6.6)	15 (9.9)
Some college	117 (40.5)	49 (32.2)
College degree	116 (40.1)	67 (44.1)
Some graduate	12 (4.2)	8 (5.3)
*Pet Owner*		
Yes	212 (73.4)	128 (84.2)
No	77 (26.6)	24 (15.8)
*Consumes meat*		13 (8.6)
Yes	--^b^	144 (94.7)
No	--	8 (5.3)
*Wild game meat consumption*^c^		
< Once per week	--	136 (89.5)
1–2 per week	--	5 (3.3)
3–4 per week	--	2 (1.3)
5–6 per week	--	1 (< .1)
Every day	--	0 (0)

^a^Respondent percentages are *n* / total participants, including incomplete data. All other percentages are *n* / participants who completed the study.

^b^Dashes reflect that Study 1 did not ask about meat consumption.

^*c*^Wild game consumption percentages add to less than 100% because vegetarians (5.3%) were not asked that question.

#### Questionnaire design

The study materials consisted of an electronic survey containing sections on demographics, bite reporting intentions, and species-to-species disease transmission beliefs. Demographics questions included sex, sexual orientation, ethnicity, education level, language(s) spoken, and pet ownership.

In the bite reporting section, participants were asked to judge their likelihood of reporting bites from various target animals to a health professional. Participants were told that a health professional could include anything from a doctor or a nurse to a health advice hotline. Participants judged likelihood of reporting for each animal using a slider that could be adjusted in units of 1 from 0–100. The numeric scale also included descriptive labels (*Very Unlikely*, *Unlikely*, *Somewhat Unlikely*, *Undecided*, *Somewhat Likely*, *Likely*, and *Very Likely*) presented above the slider to facilitate consistent use of the scale. Mammal and bird reporting were presented in a random order on separate pages. Mammals included dogs, skunks, monkeys, bats, and squirrels. Birds included grackles, swans, robins, blue jays, and peacocks.

The species-to-species disease transmission beliefs section employed the same sliding scales as the bite reporting section, but participants were asked to rate the likelihood of between-animal disease transmission for a hypothetical new disease. For each question, participants were told, “Scientists discover that a new disease can infect the liver tissue of [premise animal]. How likely is it that this disease can infect the following animals: [conclusion animals]?” The conclusion animals were listed on separate lines with individual scales (0–100) after the premise prompt. Premise animals included bats, dogs, skunks, monkeys, grackles, blue jays, swans, peacocks. Conclusion animals included bats, dogs, skunks, monkeys, squirrels, grackles, robins, blue jays, swans, and peacocks. Fewer premise animals were used than conclusion categories so that less time would be required to complete the survey and to reduce participant attrition. Animals only appeared as conclusion categories when they were not the premise animal. Premises were presented in a random order on separate pages.

#### Statistical analysis

Our primary goal was to test how mean intentions to report bird and mammal bites related to beliefs about mammal-to-mammal, bird-to-bird, and between bird and mammal disease transmission. To this end, we calculated subject means for each of these variables. Cronbach’s *α* was used to test whether responses to different mammal and bird questions were reliable within subjects, which is a prerequisite to averaging across the individual questions. To test how mean bite reporting measures were associated with mean beliefs about interspecies disease transmission, we present the associations using Kendall’s tau (τ), a non-linear correlation appropriate for ordinal data. However, the results are qualitatively identical with standard linear correlations.

To test whether mean bite reporting and interspecies disease transmission varied across any of the animal species, we used linear mixed effects models, implemented in R’s nlme package [27]. Random effect terms included random intercepts and random species effects for participants. Omnibus F-tests were used to assess whether there was significant variance between species on any measure, which were followed by paired samples t-tests to reveal the direction of the effects. Effect sizes for the F-tests are reported as partial eta-squared, and effect sizes for the t-tests are reported as Cohen’s *d*.

To test whether any demographic variables influenced the primary associations reported above, we ran a series of multiple linear regression models examining whether the effect of between bird and mammal disease transmission ratings on bird bite reporting or mammal-to-mammal disease transmission ratings on mammal bite reporting varied as a function of the following demographics: age, sex, pet ownership, education, and political orientation. We did not test ethnicity and sexual orientation as potential moderators because of the low number of respondents who were non-white or non-heterosexual. Likewise, because of low numbers of respondents in many categories for education, we coded education as a binary factor based on whether the respondent had received a college degree (0 = No college degree, *n* = 136; 1 = College degree, *n* = 153). For political orientation, we treated the spectrum from liberal to conservative as a linearly spaced continuous variable.

### Results

**Fig 2:**
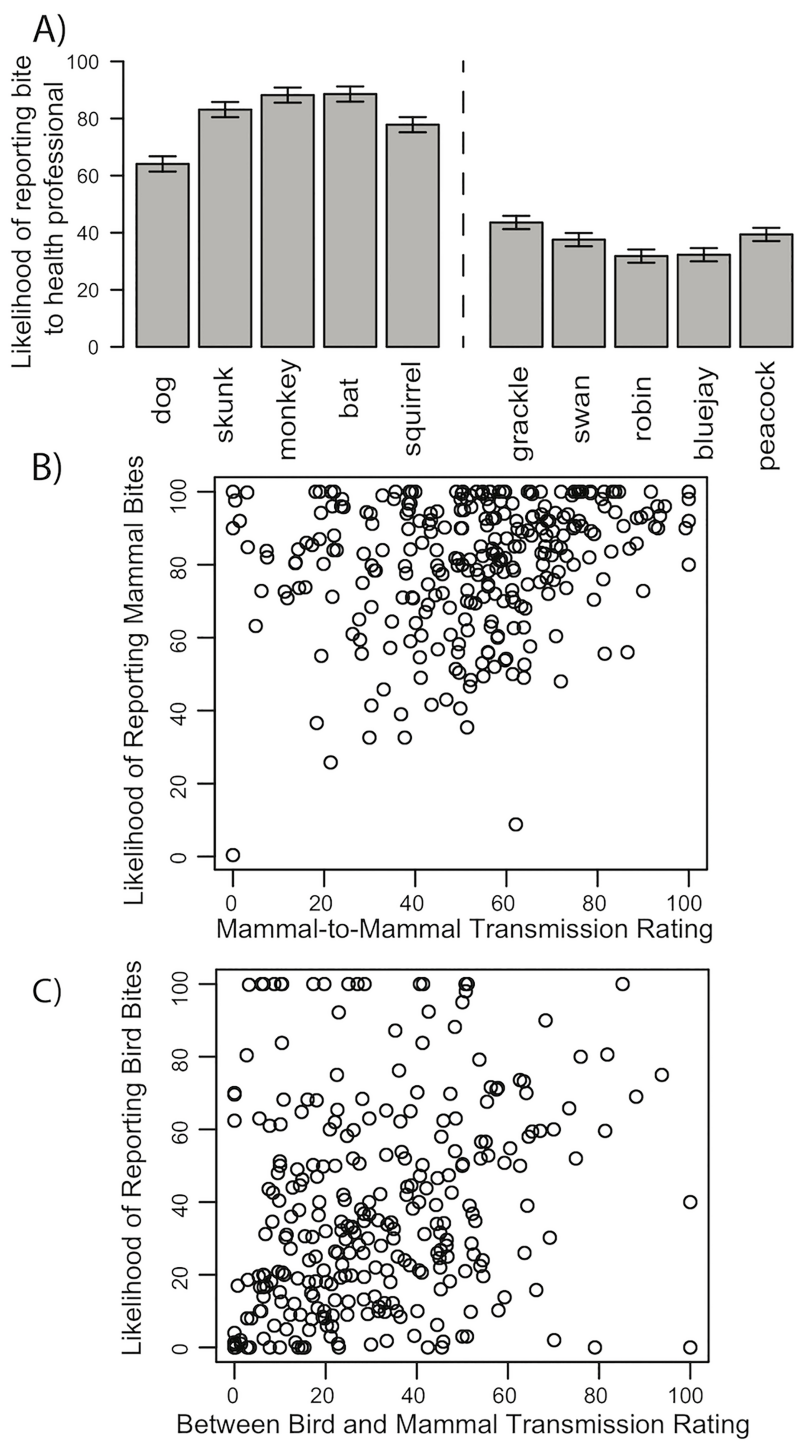
Intentions to report animal bites and associations with transmission ratings. (A) Intentions to report animal bites. (B) Association between intentions to report mammal bites and mammal-to-mammal disease transmission ratings. (C) Association between intentions to report bird bites and between bird and mammal disease transmission ratings. Error bars reflect 95% within-subject confidence intervals.

**Fig 3:**
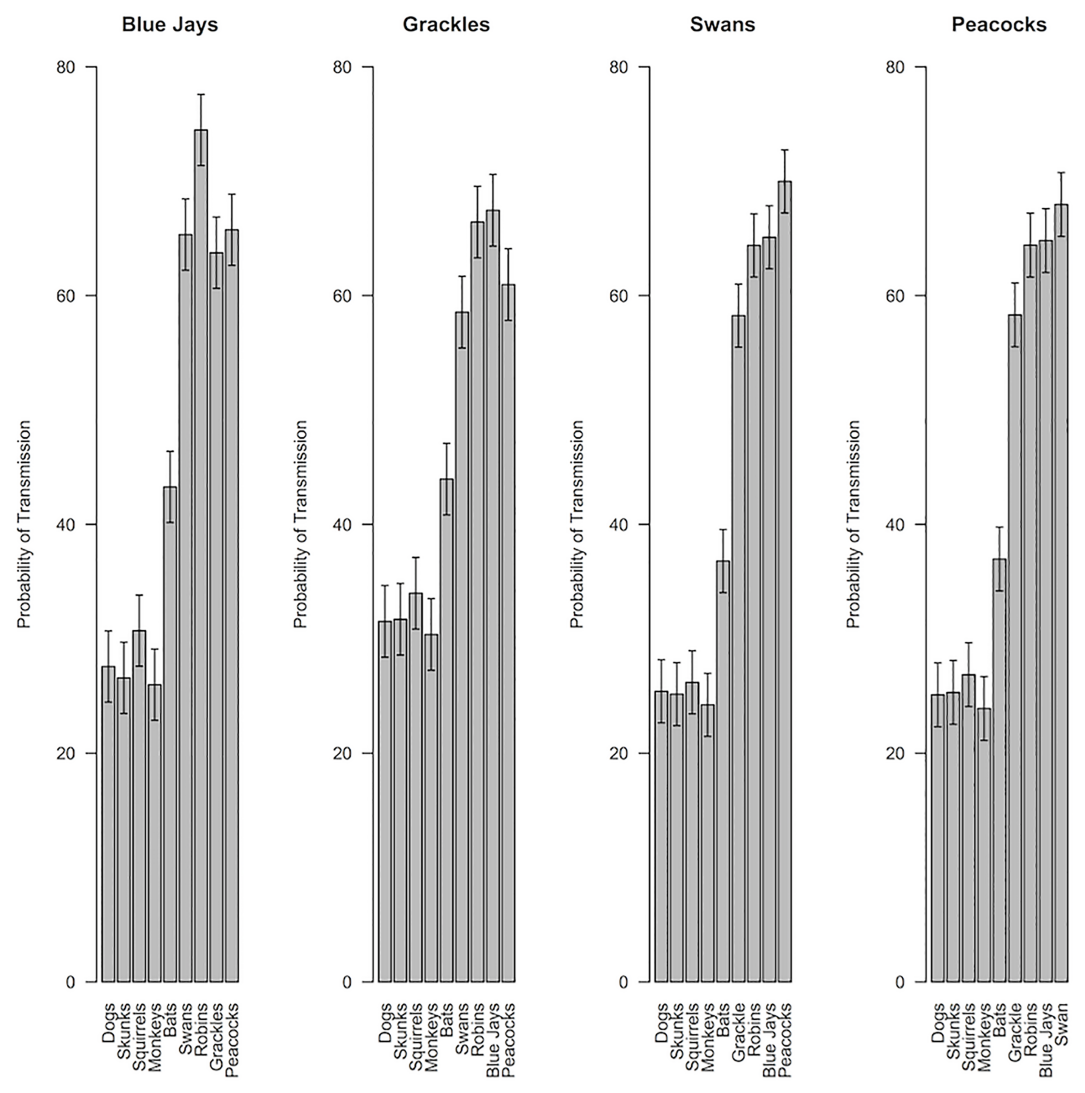
Subjective probabilities of bird disease transmission. Subjective probabilities of disease transmission between each premise animal and conclusion animal for bird premise categories. Graphs are separated by premise category. The bars reflect different conclusion categories. Thus, the first bar in the first graph is the mean perceived likelihood of a blue jay disease being transmitted to a dog. Error bars represent 95% confidence intervals calculated from separate linear mixed effects models examining how species-to-species disease transmission beliefs varied for each of the premise birds.

**Fig 4:**
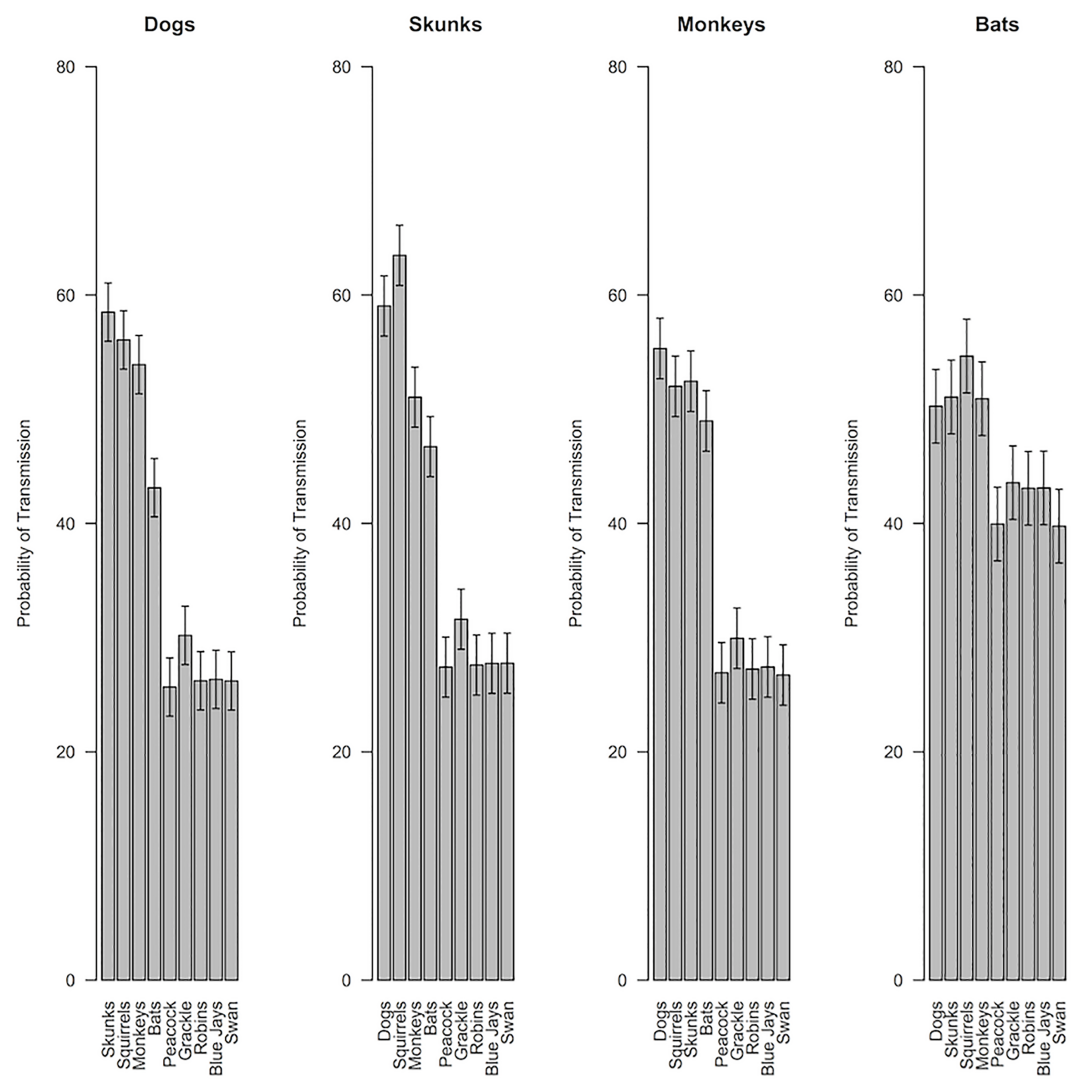
Subjective probabilities of mammal disease transmission. Subjective probabilities of disease transmission between each premise animal and conclusion animal for mammal premise categories. Graphs are separated by premise category. The bars reflect different conclusion categories. Thus, the first bar in the first graph is the mean perceived likelihood of a dog disease being transmitted to a squirrel. Error bars represent 95% confidence intervals calculated from separate linear mixed effects models examining how species-to-species disease transmission beliefs varied for each of the premise mammals.

Intentions to report mammal and bird bites were highly reliable within person (Mammals: Cronbach’s *α* = 0.86; Birds: *α* = 0.95), as were judgments of interspecies disease transmission (Mammal-to-mammal: *α* = 0.96; Bird-to-bird: *α* = 0.97; Between birds and mammals: *α* = 0.99). Nonetheless, linear mixed effects models revealed that intentions to report bites varied considerably between different animal species [Mammals: *F*(4,1152) = 111.1, *p* < .001, *η*_*p*_^2^ = 0.28; Birds: *F*(4,1152) = 35.23, *p* < .001, *η*_p_^2^ = .11; Fig 2A] and ratings of interspecies disease transmission varied between the different premise types [mammal-to-mammal, bird-to-bird, between birds and mammals; *F*(2,576) = 356.3, *p* < .001, *η*_*p*_^2^ = 0.55; see Figs 3 and 4 for individual premise effects]. Intentions to report bites were stronger for mammals than for birds [*t*(288) = 27.06, *p* < .001, *d* = 1.59], and diseases were rated as more likely to be transmissible within mammals or birds than between mammals and birds [Mammal-to-mammal vs. between birds and mammals, *t*(288) = 18.77, *p* < .001, *d* = 1.10; Bird-to-bird vs. between birds and mammals, *t*(288) = 23.42, *p* < .001, *d* = 1.38]. Consistent with previous research suggesting bats are viewed as similar to both mammals and birds [28], bats were rated as more likely to be susceptible to diseases from birds [*t*(288) = 13.42, *p* < .001, *d* = 0.79] and less likely to be susceptible to diseases from mammals [*t*(288) = 7.03, *p* < .001, *d* = 0.41] than were other mammals.

For our primary hypotheses about the association between interspecies disease transmission judgments and bite reporting intentions, we found that individual differences in endorsement of bird-to-bird and mammal-to-mammal disease transmission were both positively associated with individual differences in intentions to report mammal bites [Mammal-to-mammal: Kendall’s τ = .147, *p* < .001 (Fig 2B); Bird-to-bird: τ = .140, *p* < .001; Between birds and mammals: τ = .009]. Consistent with the premise number principle, endorsing greater likelihood of interspecies disease transmission was associated with stronger intentions to report mammal bites. For bird bites, only ratings of disease transmission between birds and mammals were associated with reporting intentions [Mammal-to-mammal τ = .043, Bird-to-bird τ = .077, Between birds and mammals τ = .219, *p* < .001 (Fig 2C)]. Coupled with weaker intentions to report bird bites overall, these results suggest that participants may only judge birds as risky (in terms of zoonosis) to the extent they believe birds and mammals can share diseases.

#### Exploratory analysis of demographic moderators

For the relationship between mammal bite reporting and mammal-to-mammal disease transmission, there were no moderating effects of age [*F*(1,285) = 0.13, *p* = .72, *η*_*p*_^2^ = .0005], sex [*F*(1,285) = 0.77, *p* = .38, *η*_*p*_^2^ = .003], pet ownership [*F*(1,285) = 0.0002, *p* = .99, *η*_*p*_^2^ < .0001], or political orientation [*F*(1,285) = 2.55, *p* = .11, *η*_*p*_^2^ = .009]. However, there was a marginally significant moderating effect of education, *F*(1,285) = 3.12, *p* = .079, *η*_*p*_^2^ = .01, whereby the slope relating mammal-to-mammal disease transmission beliefs and mammal bite reporting was larger for those with a college degree (*B* = 0.16 for those with a college degree vs. *B* = 0.08 for those without). This result should be interpreted with caution given the large number of moderators tested; however, it suggests the relationship between mammal-to-mammal disease transmission beliefs and mammal bite reporting may increase with education.

For the relationship between bird bite reporting and bird-to-mammal disease transmission, we did not find any significant moderating effects of age [*F*(1, 285) = 0.67, *p* = .42, *η*_*p*_^2^ = .002], sex [*F*(1,285) = 0.68, *p* = .41, *η*_*p*_^2^ = .002], pet ownership [*F*(1,285) = 1.01, *p* = .32, *η*_*p*_^2^ = .004], education [*F*(1, 285) = 0.38, *p* = .53, *η*_*p*_^2^ = .001], or political orientation [*F*(1, 285) = 1.50, *p* = .22, *η*_*p*_^2^ = .005].

### Discussion

The results of Study 1 suggest that inductive reasoning principles underlie people’s perceptions and generalizations of zoonosis risk. The greater the perceived likelihood of interspecies disease transmission, the more individuals generalized this risk to humans by expressing stronger intentions to report animal bites. However, because the results are correlational, it is difficult to infer the causal direction between the beliefs about interspecies disease transmission and bite reporting. It is possible, for example, that both interspecies disease transmission and bite reporting ratings are influenced by a common underlying factor, such as individual differences in beliefs about contagion [29] or risk attitudes [30]. Moreover, because the results examine individual differences, it is not clear whether such inductive reasoning principles could be harnessed to influence people’s beliefs about the risks associated with animal contact.

## Study 2

The goal of Study 2 was to experimentally test whether it is possible to influence people’s perceptions and generalizations of zoonosis risk through framing communications to convey a greater range of animals as being susceptible to a disease. As a case study, communications about Ebola virus vary in terms of how they describe the range of susceptible animals. The Center for Disease Control’s (CDC) factsheet [31] lists contact with fruit bats and nonhuman primates (apes and monkeys) as sources of human Ebola infection. Contrastingly, the World Health Organization’s (WHO) factsheet [32] lists chimpanzees, gorillas, fruit bats, monkeys, forest antelope, and porcupines. According to the premise diversity principle, the WHO’s factsheet should lead to stronger perceptions of Ebola risk from animal contact because it lists a broader range of animals as sources of human Ebola infection. In Study 2, to test whether communications with higher premise diversity would lead to stronger generalization to other animals that were not listed and greater perceptions of human risk, we gave participants two different communications about Ebola derived from the WHO and CDC factsheets. These communications were tailored from the published factsheets to control all other differences.

### Method

Participants were a new sample of 152 adults from the same Mechanical Turk population as described in the previous study, recruited for the experiment between March 10, 2016 and March 18, 2016 (see Table 1 for demographics and Fig 1 for recruitment diagram). All recruitment procedures, incentives, and inclusion criteria were the same as in the previous study. The first page of the survey contained an information sheet with additional details about the study, potential risks, confidentiality, and contact information for the PI. Participants decided whether or not to participate by reading the information sheet and either proceeding with the study or leaving the study site. Agreement to participate was recorded based on whether the participant proceeded beyond the information sheet. Written consent was not required due to the exempt classification and the low risk and anonymous online nature of the survey data. The consent process and study protocols were approved by the Institutional Review Board of Texas Tech University. Participants were excluded from all analyses if they left the survey prior to its end. All survey materials, analyses, and data are available at http://osf.io/4r79f.

#### Experimental design

The study materials consisted of an electronic survey containing a demographics section, an experimentally manipulated reading prompt about Ebola derived from the online CDC and WHO factsheets, an Ebola susceptibility section, a bite reporting intentions section, and a meat safety section. Demographics questions were the same as in Study 1, except for additional questions about meat consumption. Participants reporting that they eat meat were asked additional questions on how often they eat meat, how often they would like to eat meat, and how often they eat wild game meat. If participants answered that they wanted to eat a different amount of meat than they currently do, they were further asked to rate how much personal appearance, ethics, environment, health, spouse’s desires, cost, availability, and taste impacted this discrepancy.

For the reading prompt, participants were given the following description about Ebola and asked to fill in a blank box by detailing the animals mentioned in the description:

*The Ebola virus causes an acute, serious illness which is often fatal if untreated. Ebola virus disease (EVD) first appeared in 1976 in 2 simultaneous outbreaks, one in what is now, Nzara, South Sudan, and the other in Yambuku, Democratic Republic of Congo. The latter occurred in a village near the Ebola River, from which the disease takes its name. Ebola is introduced into the human population through close contact with the blood, secretions, organs, or other bodily fluids of infected animals such as [animal 1], [animal 2], [animal 3], and [animal 4]*.

The animals listed in the description (animals 1–4) were experimentally manipulated between participants. Participants were randomly assigned to read either a CDC-inspired set of animals (fruit bats, gorillas, monkeys, and chimpanzees; *n* = 81) or a WHO-inspired set of animals (fruit bats, monkeys, forest antelope, and porcupines; *n* = 70).

To verify that these prompts did indeed differ in their premise diversity, we had a separate group of subjects (*N* = 53) from the same Mechanical Turk population provide pairwise similarity judgments between each of the premise categories [13]. Consistent with our expectations, participants judged the CDC prompt animals to be significantly more similar to each other (i.e., less diverse), *t*(52) = 14.56, *p* < .001.

Next participants completed the Ebola susceptibility questionnaire. For each question, participants were asked, “How likely is it that [animal] can get Ebola?” (1 = *Very Unlikely*, 7 = *Very Likely*). Animals included both mammals and birds: bats, monkeys, zebras, meerkats, anteaters, giraffes, gazelles, storks, flamingos, cranes, vultures, and parrots.

Next participants completed the bite reporting questionnaire. For the bite reporting questionnaire, participants were told to *“imagine that you are on a safari and get bitten by an animal*, *but the bite just barely breaks the skin”* when considering whether they would report a bite to a health professional. Each question asked them to rate, *“How likely would you be to report being bitten by a [animal]*?*”* (1 = *Very Unlikely*, 7 = *Very Likely*).

Last, participants completed the meat safety questionnaire. For the meat safety questionnaire, participants were asked to rate, “*How safe you think it is for people in general to eat meat from each animal”* (1 = *Very Unsafe*, 7 = *Very Safe*) and to “*consider only immediate health risks from disease transmission*.*”*

#### Statistical analysis

Our primary goal was to test whether mean generalization of Ebola susceptibility to birds and mammals, mean bite reporting, and mean meat safety were affected by the wording condition (WHO or CDC-inspired). To this end, we calculated subject means for each of these variables. Cronbach’s *α* was used to test whether responses to different mammal and bird questions were reliable within subjects, which is a prerequisite to averaging across the individual questions. We used independent samples t-tests to evaluate whether the mean variables differed across wording conditions, with effect sizes reported as Cohen’s *d*.

To test whether mean Ebola susceptibility, bite reporting, and meat safety varied across any of the animal species, we used linear mixed effects models, implemented in R’s nlme package [27]. Random effect terms included random intercepts and random species effects for participants. Omnibus F-tests were used to assess whether there was significant variance between species on any measure, which were followed by paired samples t-tests to reveal the direction of the effects. Effect sizes for the F-tests are reported as partial eta-squared, and effect sizes for the t-tests are reported as Cohen’s *d*.

In addition to our primary analysis, we tested whether the effect of word condition on bite reporting and meat safety measures was mediated by increases in generalization of Ebola susceptibility. To this end, we followed the traditional Baron and Kenney [33] steps for mediation using multiple linear regression. A non-parametric bootstrapping procedure was used to test the significance of the indirect pathways between the wording condition and reporting/safety measures via the Ebola susceptibility measures [34].

To test whether any demographic variables influenced the primary associations reported above between our outcome variables (bite reporting for mammals and birds, and meat safety) and wording condition (CDC and WHO), we ran a series of multiple regression analyses using the same statistical procedures, demographic variables, and variable coding as described for Study 1.

### Results

**Fig 5:**
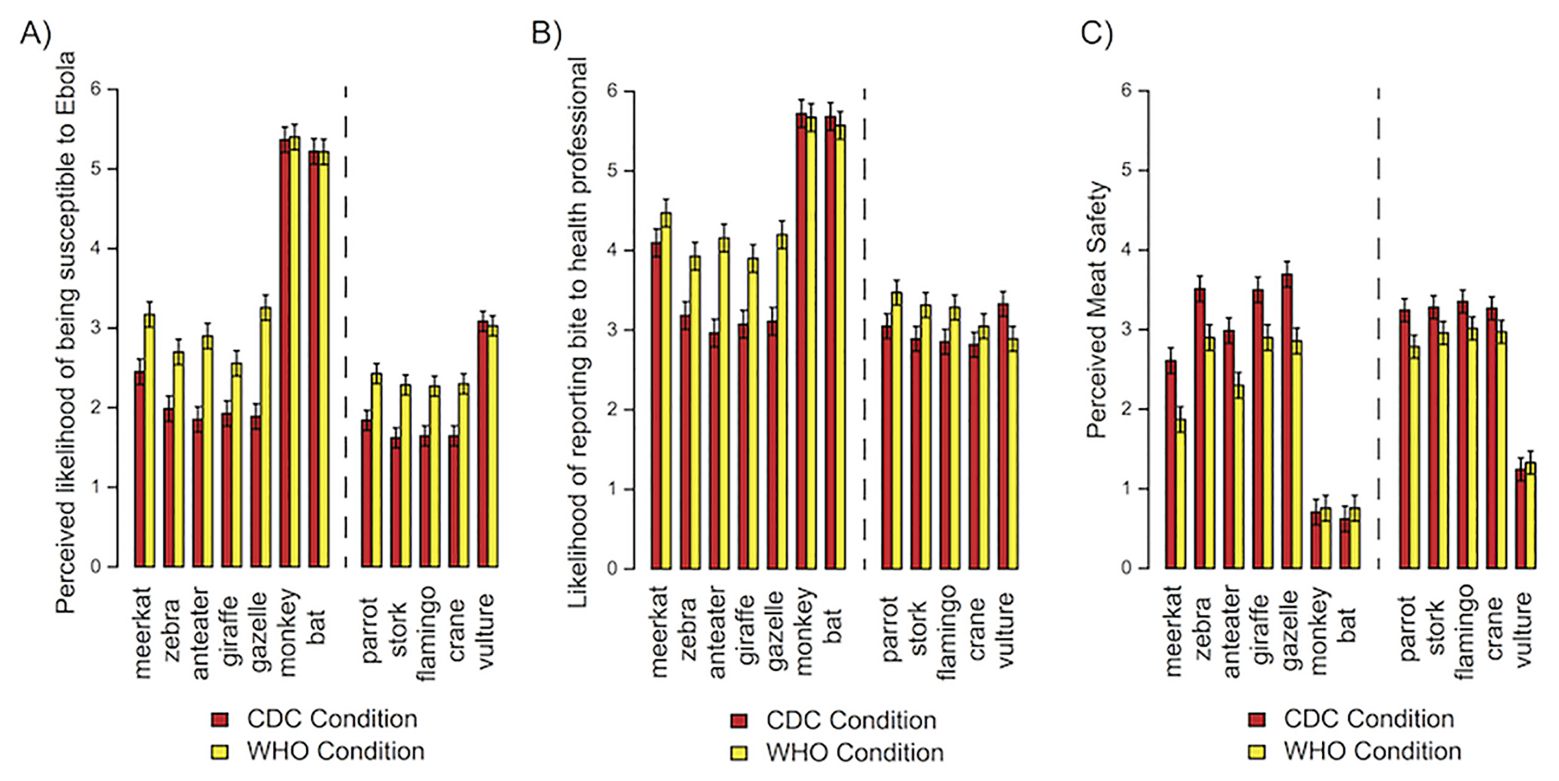
Study 2 results. (A) Perceived susceptibility of animals to Ebola. (B) Intentions to report animal bites (C) Perceived meat safety. Error bars reflect 95% within-subject confidence intervals.

The results were consistent with predictions based on the premise diversity principle. Participants in the WHO (diverse) wording condition (fruit bats, monkeys, antelopes, and porcupines) rated individual mammals as more susceptible to Ebola [*t*(150) = 3.70, *p* < .001, *d* = 0.6; Fig 5A], were more likely to report mammal bites [*t*(150) = 2.83, *p* = .005, *d* = 0.46; Fig 5B], and perceived mammal meat as less safe [*t*(150) = 2.66, *p* = .009, *d* = 0.43; Fig 5C]. Because some of the animal premises (monkeys and bats) were included in both prompts, and thus the effect of condition may have a reduced effect on ratings of these animals, we used linear mixed effects models to test whether there were interactions between condition and animal for each of our ratings. There was a significant interaction for the effect of wording condition on susceptibility ratings [*F*(6,900) = 9.38, *p* < .001, *η*_*p*_^2^ = .06], whereby the effect of condition was not significant for bats and monkeys [*t*(150) = .1, *d* = 0.02] but was significant for animals that were not included in the instructions [*t*(150) = 3.88, *p* < .001, *d* = 0.63]. There were qualitatively identical interactions between wording condition and mammals for bite reporting and meat safety [Bite reporting: *F*(6,900) = 8.58, *p* < .001, *η*_*p*_^2^ = .05; Meat safety: *F*(6,900) = 5.74, *p* < .001, *η*_*p*_^2^ = .04].

The WHO (diverse) wording condition also increased perceptions of birds’ susceptibility to Ebola [*t*(150) = 2.06, *p* = .040, *d* = 0.33] but did not significantly increase intentions to report bird bites [*t*(150) = 1.10, *d* = 0.18] or lower perceptions of meat safety [*t*(150) = 1.28, *d* = 0.21]. Like ratings of mammal susceptibility to Ebola, there was a significant interaction [*F*(4,600) = 5.76, *p* < .001, *η*_*p*_^2^ = .04], whereby there was no effect of condition for vulture susceptibility ratings [*t*(150) = 0.19, *d* = 0.03], but there was an effect for other birds [*t*(150) = 2.55, *p* = .01, *d* = 0.42]. This was not expected a priori and may have to do with vultures’ tendency to eat carrion, making perceptions of their overall exposure to Ebola higher. This interaction was also present for bite reporting, *F*(4,600) = 4.06, *p* = .003, *η*_*p*_^2^ = .03, but did not reach significance for meat safety ratings, *F*(4,600) = 1.95, *p* = .10, *η*_*p*_^2^ = .01.

In addition to our primary tests of the effect of wording condition, we used linear regression to test whether the effect of wording condition on bite reporting and perceptions of meat safety was mediated by its effect on Ebola susceptibility ratings [33]. First, we found that Ebola susceptibility ratings were significantly associated with bite reporting and meat safety perceptions for both mammals and birds, even after taking into account the effect of wording condition [Mammal bites: standardized *b* = 0.47; *t*(149) = 6.40; *p* < .001; Mammal meat: standardized *b* = -0.43; *t*(149) = 5.67, *p* < .001; Bird bites: standardized *b* = 0.51; *t*(149) = 7.23, *p* < .001; Bird meat: standardized *b* = -0.45; *t*(149) = 6.01, *p* < .001)]. Next, we found that including Ebola susceptibility in the regression model with the effect of wording condition made the effect of condition non-significant for all models [Mammal bites: *b* = 0.18; *t*(149) = 1.20; Mammal meat: *b* = -.18; t(149) = -1.16; Bird bites: *b* = .009; *t*(149) = 0.063; Bird meat: *b* = -0.06; *t*(149) = 0.43], suggesting that the effects of condition on meat safety and bite reporting were fully mediated by the effect of the different wordings on participants’ perceptions of Ebola susceptibility. A bootstrapping procedure was used to test whether the indirect pathways between condition and bite reporting and condition and meat safety ratings through perceptions of Ebola susceptibility were significantly different from zero [34].

None of the 95% bias-corrected confidence intervals included zero, suggesting that there were significant indirect pathways between wording condition and bite reporting and meat safety for both mammals and birds [Mammal bite reporting: 0.27, bias-corrected 95% CI (0.12, 0.49); Mammal meat safety: -0.25, bias-corrected 95% CI (-0.46, -0.11); Bird bite reporting: 0.17, bias-corrected 95% CI (0.003, 0.348); Bird meat safety: -0.15, bias-corrected 95% CI (-0.334, -0.012)]. Altogether, these analyses suggest that the effect of wording condition increased perceptions of disease transmission risk (through bites or wild game meat) by increasing the diversity of animals participants believe to be susceptible to Ebola.

#### Exploratory analysis of demographic moderators

For the relationship between the mammal outcome measures (bite reporting and meat safety perception) and wording condition (WHO vs CDC), we did not find any significant moderating effects of age [Bites: *F*(1,148) = 0.04, *p* = .84, *η*_*p*_^2^ = .0003; Meat: *F*(1,148) = 0.50, *p* = .48, *η*_*p*_^2^ = .003], sex [Bites: *F*(1,148) = 0.005, *p* = .94, *η*_*p*_^2^ < .0001; Meat: *F*(1,148) = 1.68, *p* = .20, *η*_*p*_^2^ = .01], pet ownership [Bites: *F*(1,148) = 0.11, *p* = .74, *η*_*p*_^2^ = .0007; Meat: *F*(1,148) = 1.07, *p* = .30, *η*_*p*_^2^ = .007], education [Bites: *F*(1,148) = 0.76, *p* = .38, *η*_*p*_^2^ = .005; Meat: *F*(1,148) = 0.18, *p* = .67, *η*_*p*_^2^ = .001], or political orientation [Bites: *F*(1,148) = 1.28, *p* = .26, *η*_*p*_^2^ = .009; Meat: *F*(1,148) = 0.001, *p* = .97, *η*_*p*_^2^ < .0001].

For the relationship between the bird outcome measures (bite reporting and meat safety perception) and wording condition (WHO vs CDC), we did not find any significant moderating effects of age [Bites: *F*(1,148) = 0.007, *p* = .93, *η*_*p*_^2^ < .0001; Meat: *F*(1,148) = 0.054, *p* = .82, *η*_*p*_^2^ = .0004], sex [Bites: *F*(1,148) = 0.01, *p* = .90, *η*_*p*_^2^ = .0001; Meat: *F*(1,148) = 0.88, *p* = .35, *η*_*p*_^2^ = .005], pet ownership [Bites: *F*(1,148) = 0.09, *p* = .76, *η*_*p*_^2^ = .0006; Meat: *F*(1,148) = 1.14, *p* = .29, *η*_*p*_^2^ = .008], or political orientation [Bites: *F*(1,148) = 0.13, *p* = .72, *η*_*p*_^2^ = .009; Meat: *F*(1,148) = 0.04, *p* = .83, *η*_*p*_^2^ = .0003]. Education was a significant moderator of the relationship between bird bite reporting and wording condition, *F*(1,148) = 4.13, *p* = .04, *η*_*p*_^2^ = .027, but was not a significant moderator of meat safety and wording condition, *F*(1,148) = 0.52, *p* = .47, *η*_*p*_^2^ = .004. Specifically, for individuals with college degrees, there was no difference between the WHO and CDC conditions in bird bite reporting intentions [WHO = 5.24; CDC = 5.44; *t*(86) = 0.56, *p* = .57]. However, bird bite reporting intentions were stronger in the WHO than the CDC condition for people without college degrees [WHO = 5.93; CDC = 4.97; *t*(62) = 2.09, *p* = .04]. Although these results are tentative given the large number of moderators tested, they may reflect a use of background knowledge that Ebola is not known to affect birds in the more highly educated group.

## General discussion

Results from both studies indicate that cognitive research on induction and generalization may be used to help combat emerging zoonoses. Although rarely studied in a cognitive framework within the zoonosis literature, humans’ inferences about risk are central to their interactions with potential disease vectors. We found that cognitive principles related to premise number and diversity impact individuals’ perceptions of zoonotic disease transmission risk and associated health behaviors. To the extent that people believe that it is possible for many diverse species to transmit diseases to one another, they generalize these risks to other species and become more wary of their own risk of infection from adverse contact with animals and consumption of animal meat.

An experiment based on CDC and WHO Ebola virus factsheets further revealed that inductive reasoning principles can be harnessed to increase perceptions of disease risk and generalization to animals not listed in the original communication. Through such cognitive framing strategies, it may be possible to reduce adverse contact with animals and increase rapid reporting of potential disease exposure. Such interventions may be particularly useful for communities in remote areas that are difficult to reach with other interventions. These results have the potential to contribute to the recent One Health initiative goals of identifying low-cost strategies for reducing emerging disease risk before outbreaks occur [35, 36].

The present results have implications for how public health practitioners approach zoonosis risk perception, particularly in terms of how communications and public health bulletins are crafted. As we found here, conveying a larger number or range of species as a potential source of a disease may increase the perception that other animals not listed could also transmit the disease, and may also increase the perceived human risk of contact. Although this seems like it may be desirable, there are cases where such generalizations and increases in human risk perception may not be a goal of public health communications. For example, one potential concern about increased perceptions of risk is that people may retaliate or behave negatively toward known reservoirs of diseases, such as bats [9]. This retaliation can have negative ecological and human impacts, as many species, like bats, may be associated with high zoonosis risk but also play central roles in agriculture as pollinators or help reduce zoonosis risk as regulators of mosquito populations [37]. Thus, communications need to be crafted in a way that encourages appropriate response to zoonotic threat, and should not be automatically aimed at promoting the strongest generalization to other species or humans without appropriate consideration of the potential downsides of such generalizations.

A key question for future research is how public health communications can incorporate knowledge of inductive reasoning strategies to increase risk perception in specific species. Here we focused on how perceived risk of animal contact (via bites and game meat consumption) related to beliefs about disease transmission and susceptibility that averaged over differences amongst species. However, not all animals are associated with the same zoonosis risk, and it will be important to understand how to tailor communications to impact species selectively. For example, bats have a strong association with emerging zoonosis [4, 38, 39], and in some cases it may be useful to tailor messages to focus on bats specifically. Although bats were associated with high levels of intended bite reporting and were perceived as being unsafe to eat, participants also may have underestimated the risks bats pose to other wildlife. Indeed, participants rated disease transmission risk between bats and other mammals as lower than for more typical (flightless) mammals. Because wildlife-livestock interactions are a major driver of emerging zoonosis [40], this finding suggests that people may underestimate the risk of grazing wildlife near bat habitats.

Cognitive research on inductive reasoning offers potential strategies to encourage generalization to specific species that we are actively pursuing in follow-up research. One such strategy is to manipulate the similarity between premise (animals described as having a disease) and conclusion categories (animals we want people to generalize to). Research suggests that people generalize knowledge from known categories to new categories on the basis of perceived similarity [13, 14]. For example, people may generalize more from cattle to horses, because they are perceived as similar, than they will between lions and horses, which may be perceived as dissimilar. Consistent with this premise-conclusion similarity principle, public health communications should include animals that are similar to the animals they would like the public to generalize to. For example, as mentioned above, livestock can be a major source of novel zoonoses, and public health officials may be able to increase the extent to which producers generalize risk to specific livestock by enumerating a larger range and number of livestock that are described as sources of infection.

Another way to study more pathogen or animal-specific generalizations is to probe, in greater depth, people’s concepts of specific zoonotic diseases to see how their representations impact the generalizations they make. Although not aimed at investigating generalization per se, one recent study examined children’s concepts of intestinal parasites by examining their drawings of the parasites [41], and how these drawings related to later performance on a test of preventative practices. In addition to giving a window into potential misconceptions about a disease, drawings may provide information about what types of generalizations people will make. For example, Rivero, et al., [41] found that some children drew oral or skin-based acquisition routes, which may be indicative of which routes are most salient to the child. Salience of particular acquisition routes may ultimately influence which preventative activities people engage in. Likewise, features of the host (animal or human) may give information on how they will generalize susceptibility to novel hosts. For example, do people who depict animal hosts (e.g., dogs) make stronger generalization of disease susceptibility to similar animals? Future research may benefit from including drawings of zoonotic diseases or transmission routes to understand how the features that people (adults and children) depict and emphasize in their drawings influence generalization.

The samples for our studies were drawn from a population of workers from Mechanical Turk, a crowd-sourcing platform that has become a key source of data for social [24] and cognitive psychology [25] experiments, as well as clinical populations [26]. Based on recapture methods, this population has been estimated to contain approximately 7,300 unique individuals for any given psychology lab, which leave and are replaced at a rate of approximately half per 7 months [42]. As with more basic cognitive reasoning experiments, we believe this population is useful for studying how the lay public may generalize knowledge and beliefs about disease risks in novel scenarios, in part because many likely have little experience with Ebola or zoonoses in general. However, this population differs from the samples used in many epidemiological studies of zoonosis risk perception, which tend to focus on populations who have higher risks for specific zoonotic diseases given their occupation, geographical region, or other demographic variables. Given this difference between our samples and more commonly studied samples in zoonosis risk perception, it will be critical for future research to further examine whether individual risk level, occupation, or other demographic variables may moderate the effect of inductive reasoning principles on risk perception. We anticipate that people’s personal experience with zoonosis, as opposed to pure risk level per se, may strengthen the relationship between beliefs and health intentions. Indeed, in the broader attitudes and public health literatures, many associations between attitudes and behaviors are modest in the general population and much stronger in groups with direct experience [43, 44]. Thus while many people in the Mechanical Turk population likely do not have direct experience with the Ebola virus or zoonoses in general, we would expect relationships between attitudes and health intentions to be even stronger among individuals who do.

Beyond personal experience with a particular zoonotic pathogen, we also expect that occupational variables may impact how people generalize zoonotic disease risks to humans and other animals. Basic heuristics like premise number and diversity are commonly used in members of the general population with little knowledge or experience with a given category [45–47]. Experts, on the other hand, may use more causal knowledge about the local ecology or epidemiological or biological models to reason about disease risk. For example, when judging whether a fish disease may spread to other species, expert fisherman are more likely than novices to use ecological knowledge about a species’ role in the food chain to generalize perceived risk [45]. Therefore, we expect that experts, depending on their occupation, may use direct causal knowledge about a disease more often than simple inductive heuristics when reasoning about risks in novel scenarios. Indeed, given there is currently no evidence of birds’ susceptibility to Ebola [48], we expect that veterinarians and public health experts with stronger biological backgrounds would, at the very least, use this knowledge when judging how announcements, like the WHO and CDC prompts we used in Study 2, generalize to birds. In short, we would expect some experts to discount the possibility that a stork bite would cause Ebola based solely on their knowledge of stork biology and Ebola epidemiology. Consistent with this prediction, while not experts per se, we did find that the effect of wording condition on bird bite reporting intentions was smaller for those with a college degree in Study 2, potentially reflecting use of background knowledge about bird’s susceptibility to Ebola. However, in Study 1, having a college degree was associated with a marginally stronger relationship between mammal-to-mammal disease transmission and mammal bite reporting, suggesting those with higher levels of knowledge do not wholly abandon basic inductive heuristics. It will be important for future work on zoonosis risk perception to further investigate potential differences in inductive generalizations between specific occupational groups and novices by including questions measuring occupational experts’ generalization to novel or different species, pathogens, or prophylactic behaviors on surveys.

Although we expect expert occupational groups to vary from our Mechanical Turk population in terms of their application of basic inductive heuristics, it is also important to consider how the present results may generalize to populations with *lower* rates of education. Indeed, our population was highly educated (approximately half of both samples had received college degrees), which is roughly consistent with populations targeted by communications in Western countries and in Bingham et al.’s [15] dog bite reporting study that inspired our work. However, many populations affected by zoonoses, and Ebola in particular, have lower rates of education. Given use of inductive reasoning heuristics like premise diversity begin to emerge in children during the elementary school years [49, 50], we anticipate that the present results will generalize to populations with lower levels of education. However, because many inductive reasoning theories are based off of research with fairly homogeneous groups of North American and European participants, it will be important to continue to test how the present results generalize to populations with different demographic profiles. For example, beyond education, we expect that some cultures with rich connections with their natural environments may rely, in certain contexts, on their ecological knowledge and beliefs when making generalizations, in much the same way that experts tend to rely more on causal reasoning than novices do [51, 52].

The present research is primarily aimed at using principles from cognitive psychology research on induction to inform research on how people generalize zoonosis risk. Although here we are concerned with a specific application of cognitive theory, the current results may have implications for basic psychological research on contagion as well. The law of contagion is a prominent social psychology construct that describes people’s tendencies to believe that negative (and positive) properties, including diseases and social ills, can be transmitted to objects or people through mere contact [18, 53]. Current theories of sympathetic magical thinking often make distinctions between the law of contagion and the law of similarity, a separate construct that describes the belief that objects with shared surface features also share deeper common essences (e.g., leading to disgust with fudge shaped like dog feces, and beliefs that voodoo dolls can affect the people they resemble [54]). The present results suggest that the laws of contagion and similarity may not be fully separate, and similarity-based effects may influence perceptions of contagion. Indeed, theories suggest that inductive reasoning principles like premise number and diversity can increase generalization of properties (such as disease susceptibility) via similarity-relationships between known and novel/unknown examples. For example, the diverse prompts in our second experiment may have increased perceptions of Ebola susceptibility by increasing the likelihood that the unknown examples would match the known examples in some respect. A major question in cognitive psychology is how different respects [55] in which items can be similar (e.g., sharing the same category [13], matching surface or internal properties [56, 57], or playing the same or complementary roles in a causal-relational system [58]) impact generalization of novel/unknown properties. Although our data do not distinguish between these different candidate theories for similarity-based transfer of contagion, the results are suggestive that beliefs about contagion can be transferred via such similarity relationships.

The present results also inform basic research on induction. Although research on zoonosis risk and general judgments of health risk suggest that individual differences and person-level demographic variables influence perceptions of risk [6–8], both health behaviors and individual differences in general have largely been ignored in cognitive psychological approaches to induction. To understand the influences of induction on real-world public health scenarios like zoonosis risk, it will be important to combine insights from more applied domains with cognitive research. Indeed, in both of our studies, individual differences played a role in generalization. For example, in the second study, individual differences in beliefs about interspecies disease transmission mediated the effect of condition on bite reporting and meat safety judgments. For birds, these associations were significant even when the main effect of wording condition was not. These results suggest that, in domains like disease transmission, there are key individual difference factors in risk perception that research on induction has not yet taken into account. Given that the paradigm we have developed reveals both psychometrically robust individual differences in health behavior intentions and strong effects of experimental manipulations, the present studies offer model paradigms that future experiments may use to further investigate person-by-situation interactions involving inductive reasoning principles such as premise number and diversity.

In conclusion, emerging diseases from animals pose a substantial public health threat, yet little is known about how people judge risks associated with different drivers of zoonoses and generalize their knowledge to facilitate inferences in novel situations. The present studies illustrate that basic cognitive principles related to inductive reasoning not only impact individuals’ perceptions of disease risk and associated health behaviors, but also can be harnessed for tailoring messages to modulate people’s perceptions of risks associated with emerging zoonoses.
